# Co-occurrence of Papillary and Follicular Thyroid Carcinoma in a Patient with Hodgkin’s Disease

**DOI:** 10.4274/Tjh.2012.0173

**Published:** 2013-06-05

**Authors:** Mehmet Aşık, Faruk Özkul, Hüseyin Toman, Ahmet Durmuş, İnan Anaforoğlu, Fahri Güneş, Erdem Erdem Akbal

**Affiliations:** 1 Çanakkale Onsekiz Mart University, Faculty of Medicine, Department of Endocrinology and Metabolism, Çanakkale, Turkey; 2 Numune Education and Research Hospital, Department of General Surgery, Trabzon, Turkey; 3 Çanakkale Onsekiz Mart University, Faculty of Medicine, Department of Anesthesia and Reanimation, Çanakkale, Turkey; 4 Numune Education and Research Hospital, Department of Hematology, Trabzon, Turkey; 5 Numune Education and Research Hospital, Department of Endocrinology and Metabolism, Trabzon, Turkey; 6 Çanakkale Onsekiz Mart University, Faculty of Medicine, Department of General Medicine, Çanakkale, Turkey; 7 Çanakkale Onsekiz Mart University, Faculty of Medicine, Department of Gastroenterology, Çanakkale, Turkey

**Keywords:** Hodgkin’s disease, thyroid carcinoma, Fine needle aspiration biopsy

## TO THE EDITOR

Hodgkin’s disease (HD) is a lymphoproliferative neoplasm primarily of B cell origin. The annual incidence of HD is estimated to be 1-3 cases per 100,000 people in the world [1]. Developments in multi-agent chemotherapy regimens in HD have increased the survival rates significantly. However, these patients have begun to encounter late therapy toxicities, such as secondary solid and hematologic tumors, infertility, and pulmonary and cardiac events, which lead to delayed mortality. Second solid tumors may develop long after the first HD diagnosis. However, synchronous tumors in HD occur rarely. In addition, synchronous thyroid malignancies in HD are extremely rare. To our knowledge, up to the present only a few cases of synchronous HD and thyroid malignancies have been reported. We present a HD patient who demonstrated 2 different kinds of thyroid cancer in the early period. 

A 42-year-old man was admitted to the hematology department complaining of a lump in the neck, weight loss, and night sweats. Physical examination was unremarkable other than left cervical lymphadenopathy and thyroid nodules. Laboratory tests showed normal hemogram, biochemistry, and thyroid function results. Cervical ultrasound revealed large (up to 6×4.5 cm) bilateral, multiple conglomerated lymphadenopathies in the submandibular, cervical, and supraclavicular districts. In addition, it showed 3-cm (the largest size) multiple nodules bilaterally in the thyroid lobes. An excisional biopsy of the cervical lymphadenopathy demonstrated a mixed cellularity type of HD. However, fine needle aspiration biopsy of the thyroid nodules had not been performed. With systemic work-up, the staging procedures and bone marrow aspiration biopsy were consistent with stage IIB: unfavorable. An ABVD chemotherapy protocol (adriamycin, bleomycin, vinblastine, and dacarbazine, once every 2 weeks) was begun with the patient. When response to treatment was evaluated after 3 courses of chemotherapy, the lymphadenopathies had disappeared on computerized tomography. The treatment was continued and completed with 6 cycles. When PET/CT was performed to evaluate the patient’s response to treatment, a focal uptake in the neck was seen, confined to the thyroid lobes (SUVmax: the upper part of the right lobe and left lobe of the thyroid, 10.80 and 14.35, respectively). Lymphadenopathy was not seen. The patient was given a total thyroidectomy. Pathologic examination demonstrated a mass of about 2.5 cm in size, a follicular thyroid carcinoma in the right thyroid lobe, and a micropapillary thyroid carcinoma of approximately 0.6 cm in size in the left thyroid lobe. The patient was given 100 mCi radioactive iodine therapy in the post-operative period. In post-ablation scanning there was no uptake, except in the thyroid bed. The patient continues to be followed in remission for the 3 diseases for 3 years.

For the first time in our present case, we have detected both HD and 2 different pathologies of thyroid cancer. Previously, only one case was reported in which HD and thyroid papillary cancer were seen simultaneously [2]. Our present case showed follicular thyroid carcinoma in the right thyroid lobe and micropapillary thyroid carcinoma in the left thyroid lobe. We think the reason for this is coincidence.

Choice of treatment in HD is based on the extent or stage of the disease. Stage of the disease is determined according to the Cotswolds modified Ann Arbor staging system. It is evaluated by clinical staging techniques that include physical, laboratory, and imaging tools. Combined chemotherapy is used for early-stage unfavorable HD. At present, 4-6 cycles of ABVD chemotherapy protocol are usually used for stage I-II A/B unfavorable disease, because it highly effective and has a favorable toxicity profile. 

The second malignancy emerging after therapy of HD has been mainly solid tumors. With prolonged follow-up after HD treatment, an annual incidence of second solid tumors of approximately 29 per 10,000 patients is seen [2]. The most common secondary cancers have been observed for the breast, lung, stomach, and bladder [3]. Risk factors for second cancers occurring after treatment of HD include exposure to radiotherapy and younger age. Increased risk of solid cancers is not expected for patients treated solely with chemotherapy [4]. The development of a second thyroid cancer in HD has been associated especially with radiotherapy treatment in the neck region [5]. Recently, one study revealed that radiotherapy treatment leads to an 18-fold increased risk of a second thyroid cancer compared to the general population [4]. The latent period after radiotherapy treatment is an important factor for developing a second thyroid cancer, with a mean latent period of 10-12 years [4,5].

Nodules of the thyroid are commonly encountered in clinical practice. Routine laboratory tests are usually inadequate to distinguish between benign and malignant nodules. Thyroid fine needle aspiration for cytology is the initial procedure of choice [6]. If our present patient had been evaluated by fine needle aspiration biopsy at first admission, his thyroid cancers would have been diagnosed earlier. When HD was diagnosed, we focused on the primary disease and its staging because HD rarely occurs together with a second primary thyroid malignancy. In conclusion, every patient with HD with thyroid nodules should be evaluated with a thyroid biopsy.

## CONFLICT OF INTEREST

None of authors of this paper has any conflicts of interest, including specific financial interests, relationships, and/or affiliations, relevant to the subject matter or materials included in this manuscript. 

## References

[1] Parkin DM, Whelan SL, Ferlay J, Teppo L, Thomas DB (2002). Cancer incidence in five continents. Volume VIII. IARC Sci Publ.

[2] Dudeque Pianovski MA (2003). A teenager with simultaneous Hodgkin disease and thyroid carcinoma. Med Pediatr Oncol.

[3] Ng AK, Bernardo MP, Weller E, Backstrand KH, Silver B, Marcus KC, Tarbell NJ, Friedberg J, Canellos GP, Mauch PM (2002). Long-term survival and competing causes of death in patients with early-stage Hodgkin’s disease treated at age 50 or younger. J Clin Oncol.

[4] Sklar C, Whitton J, Mertens A, Stovall M, Green D, Marina N, Greffe B, Wolden S, Robison L (2000). Abnormalities of the thyroid in survivors of Hodgkin’s disease: data from the Childhood Cancer Survivor Study. J Clin Endocrinol Metab.

[5] Dores GM, Metayer C, Curtis RE, Lynch CF, Clarke EA, Glimelius B, Storm H, Pukkala E, Leeuwen FE, Holowaty EJ, Andersson M, Wiklund T, Joensuu T, van’t Veer MB, Stovall M, Gospodarowicz M, Travis LB (2002). Second malignant neoplasms among long-term survivors of Hodgkin’s disease: a population-based evaluation over 25 years. J Clin Oncol.

[6] Mazzaferri El (1993). Management of a solitary thyroid nodule. N Engl J Med.

